# HPV E6/E7 mRNA in situ hybridization in endocervical adenocarcinoma: implications for prognosis and diagnosis

**DOI:** 10.1186/s12935-021-02349-1

**Published:** 2021-12-03

**Authors:** Rong-Zhen Luo, Shu-Lin Chen, Mei Li, Yue Li, Xia Yang, Li-Li Liu

**Affiliations:** 1 Sun Yat-Sen University Cancer Center, State Key Laboratory of Oncology in South China, Collaborative Innovation Center for Cancer Medicine, Guangzhou, 510060 China; 2 Department of Pathology, Sun Yat-Sen University Cancer Center, 651# Dong Feng Road East, Guangzhou, 510060 Guangdong China; 3 Department of Clinical Laboratory, Sun Yat-Sen University Cancer Center, Guangzhou, 510060 China; 4 Research Center for Translational Medicine, the First Affiliated Hospital, Sun Yat-Sen University, 58 Zhongshan Road 2, Guangzhou, 510080 Guangdong People’s Republic of China; 5 Department of Molecular Diagnostics, Sun Yat-Sen University Cancer Center, Guangzhou, 510060 China

**Keywords:** Endocervical adenocarcinoma, Prognosis, HPV E6/E7 mRNA in situ hybridization, Lymphovascular invasion, Lymph node involvement

## Abstract

**Background:**

Human papillomavirus (HPV) E6/E7 mRNA in situ hybridization (HPV E6/E7 RNAscope) appears to be a sensitive and specific technique in detecting transcriptionally active HPV. We aimed to examine the diagnostic utility of this technique in endocervical adenocarcinoma (ECA), to explore the prognostic factors for ECA patients and develop a clinically useful nomogram based on clinicopathological parameters to predict it.

**Methods:**

We retrospectively analyzed 200 patients with ECA who had undergone surgery at Sun Yat-sen University Cancer Center from 2010 and 2014. The diagnostic performance of HPV E6/E7 RNAscope were evaluated by receiver operating characteristic (ROC) curve. A prognostic nomogram model including HPV E6/E7 RNAscope was generated based on multivariate Cox regression analysis, then we compared the predictive accuracy of the prognostic model with FIGO staging and treatment using concordance index (C-index), time-dependent ROC (tdROC), and decision curve analysis (DCA).

**Results:**

The sensitivity and specificity of HPV E6/E7 RNAscope for distinguishing HPV-associated adenocarcinoma (HPVA) from non-HPV-associated adenocarcinoma (NHPVA) in the whole cohort were 75.8% and 80%, respectively. According the univariate analysis and multivariate logistic regression analysis, age, lymphovascular invasion (LVI), lymph node involvement (LNI), and HPV E6/E7 RNAscope were valuable predictive factors for OS. These parameters were incorporated into the nomogram model (nomogram A) compared with FIGO stage and treatment. The C-index of nomogram A for predicting OS was 0.825, which was significantly higher than FIGO stage (C-index = 0.653, p = 0.002) and treatment (C-index = 0.578, p < 0.001).

**Conclusions:**

HPV E6/E7 RNAscope is highly specific for ECA, and the 4-variable nomogram showed more accurate prognostic outcomes in patients with ECA.

**Supplementary Information:**

The online version contains supplementary material available at 10.1186/s12935-021-02349-1.

## Introduction

Endocervical adenocarcinoma (ECA) accounts for 15%–20% of all cervical carcinomas. Studies have shown that ECA is increasingly reported in young women [1], with a projected 5-year overall survival (OS) rate of only 20.3% (95% confidence interval [CI]: 14.2–27.1%) compared to the squamous type (31.3%, 95%CI: 29.2–33.3%) [2]. The International Federation of Gynecology and Obstetrics (FIGO) staging system is the gold standard for predicting outcomes in patients with ECA, and the mainstay treatment of advanced ECA is concurrent chemoradiotherapy. However, ECA patients with the same FIGO stage can have marked heterogeneities in their outcomes [3, 4]. The new FIGO 2018 amendments put forward by the gynecological oncology committee agree that staging is an ongoing process informed by data on prognosis and survival, and therefore, treatment should be individualized and not merely based on staging given the variability of the resource across regions. FIGO 2018 also recommends any imaging modality and/or pathological findings like lymphovascular invasion (LVI) for allocating staging [3].

A new classification—the International Endocervical Adenocarcinoma Criteria and Classification (IECC) has replaced the 2014 WHO classification and categorizes ECA based on the morphological features linked to the etiology (i.e., HPV infection), thus grouping them into HPV-associated (HPVA) and non-HPVA (NHPVA) types [4, 5]. Comparing HPVA with NHPVA types reveals essential differences in tumor biology and patient survival, with NHPVA-type tumors significantly associated with worse outcomes [6, 7]. It is apparent that a classification based on the pathogenesis and other clinicopathological factors may be more clinically useful and reproducible than the current FIGO scheme. The other shortcoming of FIGO is that it doesn't stratify patients based on histology and HPV status. Hence, novel prognostic determinants to complement the FIGO staging system is needed. The validity of this new classification is supported by HPV status but with limited clinical data[8, 9].

In the present studies, the following tests were used to detect HPV infection: HPV DNA, genotype, RNAscope, and immunochemistry (IHC) against p16 protein. Plasma HPV DNA levels can be determined using PCR and is a potential marker for cervical cancer [10]. The other 3 assays can be conducted on formalin-fixed paraffinembedded (FFPE) samples. Immunostaining for p16 is a cost-effective and highly sensitive marker but with a low specificity [11]. PCR for HPV genotyping is the gold-standard assay to diagnose active HPV infection [12]. Chromogenic in situ hybridization against RNA can identify HPV in situ via microscopic observation [13]. The technique is expensive but can be used on FFPE samples [14]. In situ detection of HPV E6/E7 mRNA using the RNAscope also appears to be a sensitive and a specific method; hence, we aimed to investigate the potential prognostic utility of this technique in ECAs.

Numerous controversies surround the FIGO staging system, wherefore ECAs are morphologically stratified into A, B and C subgroups using the Silva based on their LVI or lymph node involvement (LNI) status [15]. Tumors characterized by well-demarcated glands with rounded contours are designated as pattern A; tumors with early or limited, localized destructive invasion or inflamed stroma adjacent to an intact gland are designated pattern B, while tumors with more aggressive features, including diffusely infiltrative glands, usually associated with extensive, diffuse desmoplastic response are designated pattern C. Therefore, clinicians could provide precision treatment to patients with pattern C tumors. However, stratifying patients based on a single biomarker, such as LVI or LNI alone, can lead to misclassification of tumors and poor patients' prognostication, hence the two parameters should be combined [16]. There is currently no such available nomogram model for patients with ECA that combines HPV, LVI and LNI status, And other survival prediction models that follow the FIGO staging system have been insufficient.

In the present study, we aim to validate the utility of the novel RNA scope method for detecting HPV RNA in tissue samples for proper initial risk stratification of patients. We also built a nomogram that will compliment FIGO staging for prognostic classification of EAC. We hope the link between these two modalities will bring new insights into the clinical care of EAC patients.

## Patients and methods

### Sampling

The present retrospective study included 200 patients with pathologically diagnosed ECA who had been treated at the Sun Yat-sen University Cancer Center from January 2010 to December 2014. We enrolled patients with confirmed pathological diagnoses of ECA with available complete clinical data and test results. We excluded patients with systemic metastasis or with synchronous primaries and any patient with a prior cancer history. The last follow-up period was June 2020. The histological diagnoses were based on our proposal for a new ECA classification: the IECC 2017 [9], and patients were grouped into HPVA and NHPVA ECA based on HE staining and microscopic morphology.

### Immunohistochemistry (IHC)

A tissue microarray consisting of the ECA and adjacent non-tumorous tissue was constructed using a tissue array instrument (Minicoreexcilone; Minicore, UK). For each patient, hematoxylin and eosin (H&E) stained slides were examined, and at least two areas from different regions were marked for sampling. A 1.0 mm diameter tissue core was punched from the marked fields and re-embedded. FFPE ECA sections were dewaxed in xylene and graded alcohols, hydrated, and washed in PBS. After pretreatment in a microwave oven, endogenous peroxidase was blocked using 3% hydrogen peroxide in methanol for 20 min. This was followed by avidin–biotin blocking using a kit (DAKO, Germany). The slides were then incubated with antibodies for p16 (Roche, Germany), MLH1 (Roche [M1], Germany), PMS2 (Dako [EP51], Germany), MSH2 (ZA0622, Zhongshan, China), MSH6 (Roche [SP93], Germany), and Ki-67 (ZA0502, Zhongshan, China) overnight at 4 °C, washed in PBS, and incubated with biotinylated goat anti-rabbit/mouse antibodies (DAKO, Germany). The slides were developed using diaminobenzidine (DAB) and counterstained with hematoxylin. Two independent, experienced pathologists evaluated the staining; and p16 was interpreted as positive if diffuse, block-like staining was found in all cores. No staining or patchy staining was interpreted as unfavorable. MSH2/MSH6/PMS2/MLH1 was interpreted as positive if ≥ 1% of the tumor cell nuclei were positive. Representative images were show in Additional file 1: Fig. S1.

### HPV DNA

DNA load of High-risk-HPV (HR-HPV) in plasma sample was evaluated by Digene second generation hybrid capture (HC2) DNA test and in vitro acid hybridization assay with signal amplification using microplate chemiluminescence for HR-HPV DNA detection. A commercial kit detecting high risk was used. The alkaline solution was used to denature the double-stranded DNA, and the liberated single-strand DNA was combined with the RNA probe mix. RNA–DNA hybrids were then transferred to a capture microplate coated with goat polyclonal for immobilization. Subsequently, alkaline phosphatase-conjugated antibodies were added to the RNA–DNA hybrids. After washing, the chemiluminescent substrate was added for signal amplification. Results were analyzed by DIGENE DML 2000 Software. DNA load was expressed by the unit of Relative Light Units/Cutoff Value (RLU/CO), representing the ratio of the light emission of the sample to the average of three positive control samples. HR-HPV DNA load of 1.0 (pg/ml) or more was defined as HR-HPV DNA positive.

### HPV genotype

Tissues were scraped by coverslip as per the labeled region on the H&E slide and transferred into a clean 1.5 ml centrifuge tube. DNA was extracted using a QIAGEN DNA FFPE Kit (Qiagen, Dusseldorf, Germany) according to the manufacturer's instructions and was quantified with a Nano-Drop2000 (NanoDrop Technologies, Wilmington, DE, USA). HPV was detected by the Human papillomavirus Genotyping Test Kit (Anhui Dajian, Guangzhou, China) using the BioRad CFX96 real-time quantitative PCR instrument. The Roche Cobas 4800 system (Roche, Pleasanton, CA) was used to detect HPV and evaluate the presence of the following 14 types of HPV DNA: 16, 18, 31, 33, 35, 39, 45, 51, 52, 56, 58, 59, 66, and 68.

### HPV E6/E7 mRNA in situ hybridization

The stained slides of each specimen were examined using the RNAscope scoring system, as described in previous studies [17, 18]. High-risk HPV subtypes were evaluated on all ECAs in the tissue microarray that had sufficient tissue to allow scoring (n = 200). HPV fluorescent in situ hybridization (FISH) was performed using a chromogen and the RNAscope system (Advanced Cell Diagnostics; catalog No. 312598). The RNAscope probe “HPV HR18” contains probes that target E6 and E7 mRNA in the following high-risk subtypes: HPV16, 18, 26, 31, 33, 35, 39, 45, 51, 52, 53, 56, 58, 59, 66, 68, 73, and 82. RNAscope results were classified into five degrees based on the following scoring guidelines: score 0, no staining or less than one dot in every ten cells (visible at 40 × magnification); score 1, 1–3 dots per cell (visible at 20–40 × magnification); score 2, 4–10 dots per cell, with very few dot clusters (visible at 20–40 × magnification); score 3, > 10 dots per cell, with < 10% of positive cells having dot clusters (visible at 20 × magnification); score 4, > 10 dots per cell with > 10% of positive cells having dot clusters (visible at 20 × magnification). Cases with RNAscope score ≥ 1 were identified as positive.

### Data collection

Among the patients enrolled, the following variables were recorded: age, the modality of treatment (Surgery with or without chemotherapy/radiotherapy), HPV DNA, HPV subtype, HPV E6/E7 RNAscope, histological type, tumor size, differentiation, LVI, invasion level of the uterine cervix, LNI, parametrium invasion, surgical margin, MMR status, p16, Ki-67, and FIGO stage.

### Statistical analysis

Statistical investigations were conducted using SPSS 19.0 (IBM Corp., Armonk, NY, USA) and R 3.4.0 (http://www.R-project.org/). Continuous data were expressed as means ± standard deviations and were analyzed using Student’s t-test or Mann–Whitney U test, as appropriate based on the results of the Kolmogorov–Smirnov test. Categorical variables were presented using numbers (percentages) and were analyzed with Fisher’s exact test. Survival analysis was conducted utilizing the Kaplan–Meier curves. Univariate and multivariate Cox proportional hazard regression were used to explore the risk factor for OS. The best cutoff values were determined using X-tile [19]. The best cutoff values were determined using X-tile with the following results: age, 37 years; RNAscope, 3.3 (Additional file 1: Fig. S2A); tumor size, 4.5 cm, and Ki-67 (12.5%). Receiver operating characteristic curves (ROC) were generated to compare the diagnostic performance of different detection method for ECA patients. The model's predictive efficiency and discriminative capability were defined by a concordance index (C-index) and a calibration curve. A two-sided p-value < 0.05 was considered statistically significant.

## Results

### Patients’ characteristics and survival

We enrolled 200 patients with ECA for analysis, of which 185 were HPVA and 15 NHPVA (Table 1). Across both groups, the median follow-up period was 68 months (range: 1.5–125.1 months). The 1, 3, and 5-year OS rates were 99.5%, 90.5%, and 85.0%, respectively.

**Table 1:** Patient demographics and clinical charateristics

Characteristic	Total (n = 200)	HPVA (n = 185)	NHPVA (n = 15)	*P*^a^
	No. (%)	No. (%)	No. (%)	
Age				**0.014**
≤ 37	34 (17.0%)	28 (82.4%)	6 (17.6%)	
> 37	166 (83.0%)	157 (94.6%)	9 (5.4%)	
HPV DNA				** < 0.001**
Negative (0–1 pg/ml)	34 (17.0%)	26 (76.5%)	8 (23.5%)	
Positive (≥ 1 pg/ml)	110 (55.0%)	108 (98.2%)	2 (1.8%)	
Not avaiable	56 (28.0%)	51 (51.8%)	5 (4.2%)	
HPV genotype				0.273
HPV 16	49 (24.5%)	47 (95.9%)	2 (4.1%)	
HPV 18	60 (30.0%)	57 (95.5%)	3 (5.0%)	
Other	11 (5.5%)	11 (100.0%)	0 (0.0%)	
Negative	72 (36.0%)	63 (87.5%)	9 (12.5%)	
Not avaiable	8 (4%)	7 (87.5%)	1 (12.5%)	
HPV RNAscope				**0.003**
≤ 3.3	99 (49.5%)	86 (46.5%)	13 (13.1%)	
> 3.3	101 (50.5%)	99 (98.0%)	2 (2.0%)	
Tumor size (cm)				**0.004**
≤ 4.5	178 (89.0%)	168 (94.4%)	10 (5.6%)	
> 4.5	22 (11.0%)	17 (77.3%)	5 (22.7%)	
FIGO stage				** < 0.001**
I	141 (70.5%)	137 (97.2%)	4 (2.8%)	
II	51 (25.5%)	43 (84.3%)	8 (15.7%)	
III	5 (2.5%)	4 (80.0%)	1 (20.0%)	
IV	3 (1.5%)	1 (33.3%)	2 (66.7%)	
Differentiation				0.537
Good	10 (5.0%)	7 (70.0%)	3 (30.0%)	
Moderate	106 (53.0%)	96 (90.6%)	10 (9.4%)	
Poor	84 (42.0%)	82 (97.6%)	2 (2.4%)	
LVI				0.141
None (0)	138 (69.0%)	130 (94.2%)	8 (5.8%)	
Focal (1–4)	37 (18.5%)	31 (83.8%)	6 (16.2%)	
Moderate (5–9)	15 (7.5%)	14 (93.3%)	1 (6.7%)	
Extensive (≥ 10)	10 (66.7%)	10 (100.0%)	0 (0.0%)	
Invasion level of uterine cervix				**0.008**
< 1/3	54 (27.0%)	53 (98.1%)	1 (1.9%)	
1/3–2/3	61 (31.0%)	60 (96.8%)	2 (3.2%)	
≥ 2/3	84 (42.0%)	72 (85.7%)	12 (14.3%)	
Lymph nodes invasion				**0.024**
No	154 (77.0%)	146 (94.8%)	8 (5.2%)	
Yes	46 (23.0%)	39 (84.8%)	7 (46.7%)	
Parametrium invasion				0.599
No	181 (90.5%)	168 (92.8%)	13 (7.2%)	
Yes	19 (9.5%)	17 (89.5%)	2 (10.5%)	
Surgical margin				** < 0.001**
No	187 (93.5%)	177 (94.7%)	10 (5.3%)	
Yes	13 (6.5%)	8 (61.5%)	5 (38.5%)	
Treatment				0.235
Surgery	83 (41.5%)	80 (96.4%)	3 (3.6%)	
Surgery + chemotherapy	21 (10.5%)	20 (95.2%)	1 (4.8%)	
Surgery + radiotherapy	41 (20.5%)	36 (87.8%)	5 (12.2%)	
Surgery + chemoradiation	55 (27.5%)	49 (89.1%)	6 (10.9%)	
MMR status				0.978
dMMR	13 (6.5%)	12 (92.3%)	1 (7.7%)	
pMMR	187 (93.5%)	173 (92.5%)	14 (7.5%)	
P16 IHC				**0.001**
Negative	82 (41.0%)	70 (85.4%)	12 (14.6%)	
Positive	118 (59.0%)	115 (97.5%)	3 (2.5%)	
Ki-67 IHC				0.131
≤ 12.5%	48 (24%)	42 (87.5%)	6 (12.5%)	
> 12.5%	152 (76%)	143 (94.1%)	9 (5.9%)	

^a^Chi-square test. HPVA, HPV-associated adenocarcinoma; NHPVA, nonHPV-associated adenocarcinoma; IHC, immunohistochemistry; LVI, lymph vascular invasion; dMMR, delete mismatch repair; pMMR, proficient mismatch repair

### Diagnostic performance of HPV E6/E7 mRNA in situ* hybridization in ECA compared to other assays.*

**Fig. 1:**
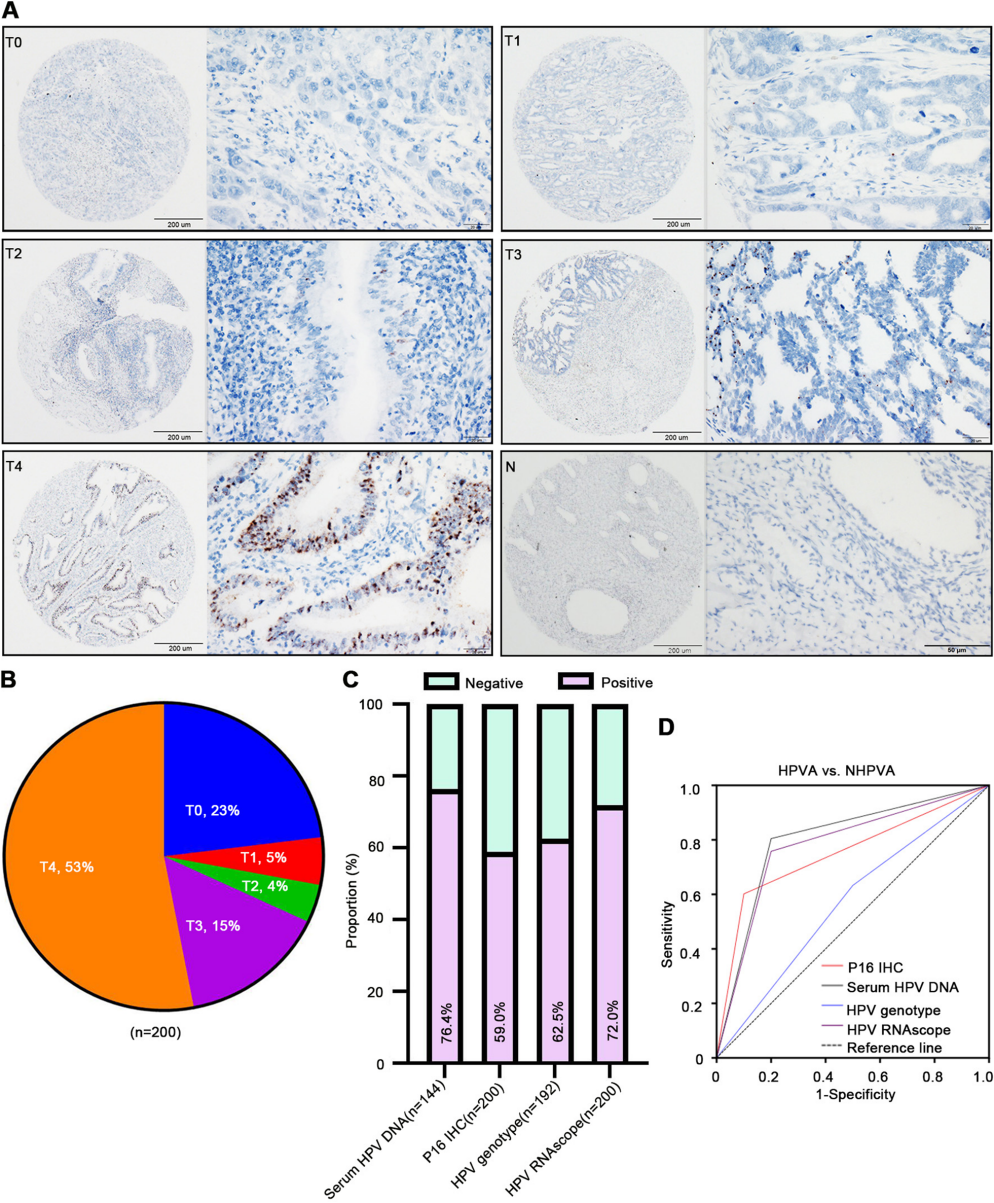
Representative images of human papillomavirus (HPV) E6/E7 RNAscope in paraffin-embedded endocervical adenocarcinoma (ECA) samples. **A** HPV mRNA was detected using RNAscope in ECA (T) and non-tumorous tissues adjacent to ECA (N). Representative images of scores 0 (T0), 1 (T1), 2 (T2), 3 (T3) and 4 (T4) are shown. **B** The proportion of RNAscope scores in ECA tissues. **C** Positive rates of HPV by PCR, RNAscope, HPV genotype and p16 IHC. (D) Receiver operating characteristics curve for HPV, detected by PCR, immunohistochemistry, genotyping and RNAscope for HPV-associated vs. non-HPV-associated types

The RNAscope scores of 0 to 4 are shown in Fig. 1A and B. The diagnostic implications of the HPV E6/E7 RNAscope scores were evaluated in 200 ECA samples that included both HPVA and NHPVA cases (Additional file 2: Table S1). The positive rates of HPV DNA, p16 IHC, HPV genotype, and HPV E6/E7 RNAscope across all ECA cases were 76.4%, 59.0%, 62.5%, and 72.0%, respectively (Fig. 1C and Additional file 1: Fig. S3). HPV RNAscope and other assays were closely related (Additional file 2: Table S2). ROC curves suggested that HPV DNA and HPV E6/E7 RNAscope showed similar results in terms of distinguishing HPVA from NHPVA (area under the curve [AUC] = 0.802, sensitivity = 80.5%, specificity = 80% vs. AUC = 0.799, sensitivity = 75.8%, specificity = 80%, respectively), outperforming both p16 IHC (AUC = 0.751, sensitivity = 60.2%, specificity = 90%) and HPV genotype (AUC = 0.566, sensitivity = 63.3%, specificity = 50%; Fig. 1D and Table 2).

**Table 2:** Diagnostic performances of studied testing for ECA patients

HPVA vs. NHPVA	AUC	95%CI	Sensitivity	Specificity
P16 IHC	0.751	0.620–0.882	0.602	0.900
HPV DNA	0.802	0.653–0.951	0.805	0.800
HPV genotype	0.566	0.379–0.754	0.633	0.500
HPV RNAscope	0.799	0.629–0.929	0.758	0.800

AUC, area under curve; 95%CI, 95% confident interval; HPVA, HPV-associated adenocarcinoma; NHPVA, nonHPV-associated adenocarcinoma; IHC, immunohistochemistry; HPV RNAscope

The correlations among serum HPV DNA level, p16 IHC, HPV genotype, and HPV E6/E7 RNAscope were also studied (Additional file 2: Table S2). A significantly higher proportion of ECA cases were positive for p16 IHC, HPV genotype, and HPV E6/E7 RNAscope in subgroups with HPV DNA levels that differed by tenfold. In patients with serum HPV DNA levels of 1–100 pg/mL, the p16 IHC positivity was similar to that of HPV genotype (60%), while those with serum HPV DNA levels of 100–10,000 pg/mL, HPV genotype positivity was similar to that of HPV E6/E7 RNAscope (93.8%). The HPV E6/E7 RNAscope positivity for the diagnosis of ECA was much higher compared to p16 IHC and HPV genotype (Additional file 1: Fig. S4A). We also elicited that HPV E6/E7 RNAscope was positive in almost all cases that were p16 IHC positive (88.5% of all cases, 89.5% of HPVA cases), only that more cases were negative by IHC and serum levels (Additional file 1: Fig. S4B). In different HPV subtypes, the positivity of RNAscope for the diagnosis of ECA was much higher compared to that of p16 IHC, but lower than HPV DNA (Additional file 1: Fig. S4C). The HPV subtypes in patients with HPVA and NHPVA are listed in Additional file 2: Table S3. In cases with RNAscope scores of 3 and 4, the HPV DNA positivity was higher than both p16 IHC and HPV genotype (Additional file 1: Fig. S4D).

### Multivariate analysis of overall survival

**Fig. 2:**
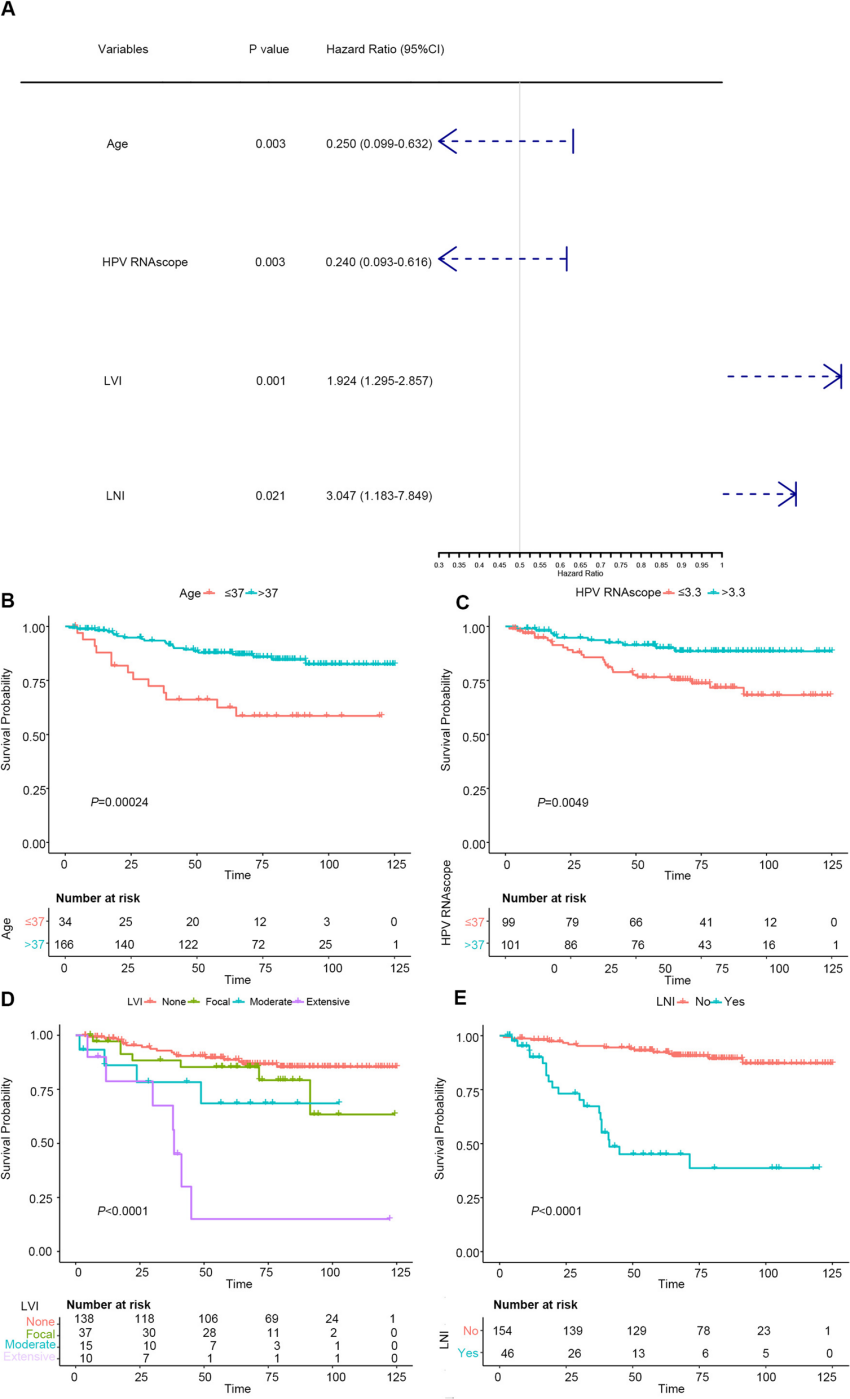
Forest plot and Kaplan–Meier curves for overall survival (OS) in patients with endocervical adenocarcinoma (ECA). **A** Forest plot showed the hazard ratio and 95% confidence interval for OS according to the Cox proportional hazards regression analysis in patients with ECA. **B**–**E** Age, human papillomavirus E6/E7 RNAscope, lymphovascular invasion, and lymph node involvement in patients with ECA in the whole cohort are plotted as a distribution

Univariate analysis indicated that the following variables were related to OS in patients with ECA: age (p = 0.002), HPV E6/E7 RNAscope (p = 0.006), tumor size (p = 0.005), FIGO stage (p < 0.001), histological type (p = 0.004), LVI (p < 0.001), invasion level of uterine cervix (p < 0.001), LNI (p < 0.001), parametrium invasion (p < 0.001), surgical margin (p = 0.006), chemotherapy (p = 0.009), radiotherapy (p < 0.001), p16 (p = 0.039), and Ki-67 (p = 0.025). In a multivariate analysis, the following items remained independently prognostic: age (HR = 0.250, 95% confidence interval [CI]: 0.099–0.632, p = 0.003), HPV E6/E7 RNAscope (HR = 0.240, 95% CI: 0.093–0.616, p = 0.003), LVI (HR = 1.924, 95% CI: 1.295–2.857, p = 0.001), and LNI (HR = 3.047, 95% CI: 1.183–7.849, p = 0.021). The results are shown in Table 3, with a forest plot in Fig. 2A. Kaplan–Meier analysis showed significant diversity (Fig. 2B–E).

**Table 3:** Univariate and multivariate Cox proportional hazards regression analysis for OS

Variables	Univariate analysis		Multivariate analysis	
	HR (95% CI)	*P* value	HR (95% CI)	*P* value
Age (≤ 37 vs. > 37 years)	0.316 (0.154–0.649)	**0.002**	0.224 (0.095–0.530)	**0.001**
HPV DNA (negative vs. positive vs. na)	0.868 (0.500–1.509)	0.616		
HPV genotype (negative vs. positive vs. na)	1.004 (0.523–1.926)	0.992		
HPV RNAscope (≤ 3.3 vs. > 3.3)	0.337 (0.156–0.730)	**0.006**	0.288 (0.124–0.673)	**0.004**
Tumor size (≤ 4.5 vs. > 4.5 cm)	3.165 (1.414–7.083)	**0.005**	0.773 (0.223–2.674)	0.684
FIGO stage (I vs. II vs. III vs. IV)	2.821 (1.703–4.672)	** < 0.001**	1.120 (0.613–2.013)	0.705
Histological type (HPVA vs. NHPVA)	0.266 (0.108–0.657)	**0.004**	1.365 (0.334–5.589)	0.665
Differentiation (good vs. moderate vs. poor)	1.435 (0.768–2.684)	0.258		
LVI (none vs. focal vs. moderate vs. extensive)	2.056 (1.508–2.803)	** < 0.001**	1.770 (1.212–2.587)	**0.003**
Invasion level of uterine cervix (1/3 vs. 1/3–2/3 vs. 2/3)	3.147 (1.754–5.648)	** < 0.001**	1.532 (0.791–2.967)	0.206
Lymph nodes invasion (no vs. yes)	9.310(4.533–19.120)	** < 0.001**	2.838 (1.162–6.931)	**0.022**
Parametrium invasion (no vs. yes)	0.231 (0.094–0.568)	**0.001**	0.696 (0.217–2.234)	0.543
Surgical margin (no vs. yes)	0.263 (0.101–0.687)	**0.006**	1.621 (0.435–6.048)	0.472
Treatment (s vs. s + ct vs. s + rt vs. s + cr)	1.733 (1.298–2.313)	** < 0.001**	1.182 (0.851–1.641)	0.318
MMR status (dMMR vs. pMMR)	1.401 (0.426–4.610)	0.579		
P16 IHC (negative vs. positive)	0.478 (0.237–0.963)	**0.039**	1.203 (0.507–2.854)	0.675
Ki-67 IHC (≤ 12.5% vs. > 12.5%)	0.446 (0.220–0.906)	**0.025**	0.841 (0.348–2.035)	0.701

*HR* hazard ratio, *CI* confidence interval, *HPVA* HPV-associated adenocarcinoma, *NHPVA* non HPV-associated adenocarcinoma, *IHC* immunohistochemistry, *LVI* lymph vascular invasion, *dMMR* delete mismatch repair, *pMMR* proficient mismatch repair, HPV genotype positive, HPV16, HPV18 and other types; s, surgery; s + ct, surgery + chemotherapy; s + rt, surgery + radiotherapy; s + cr, surgery + chemoradiation; na, not avaiable

### Construction of the prognostic nomogram model and comparison of predictive accuracy between Nomogram model, FIGO stage and treatment.

**Fig. 3:**
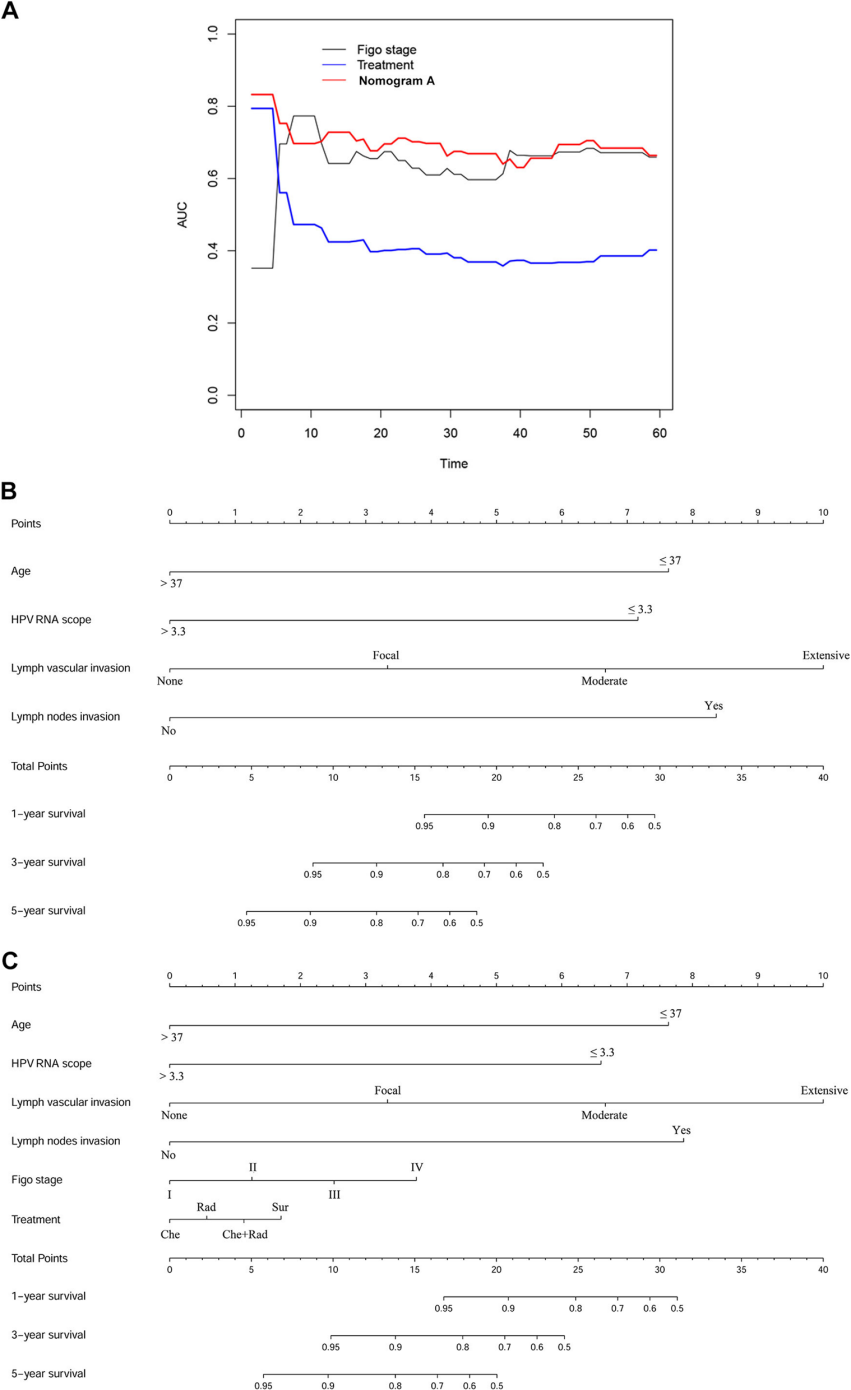
Time-dependent ROC curve analysis and nomogram model for overall survival (OS) in patients with endocervical adenocarcinoma ECA. **A** Time-dependent receiver operating characteristic curves showing the sensitivity and specificity of the nomogram model for predicting OS. **B**, **C** The nomogram models A and B were used to summate the points identified on the points scale for each variable, indicating the probability of 1-, 3-, and 5-year survival

**Fig. 4:**
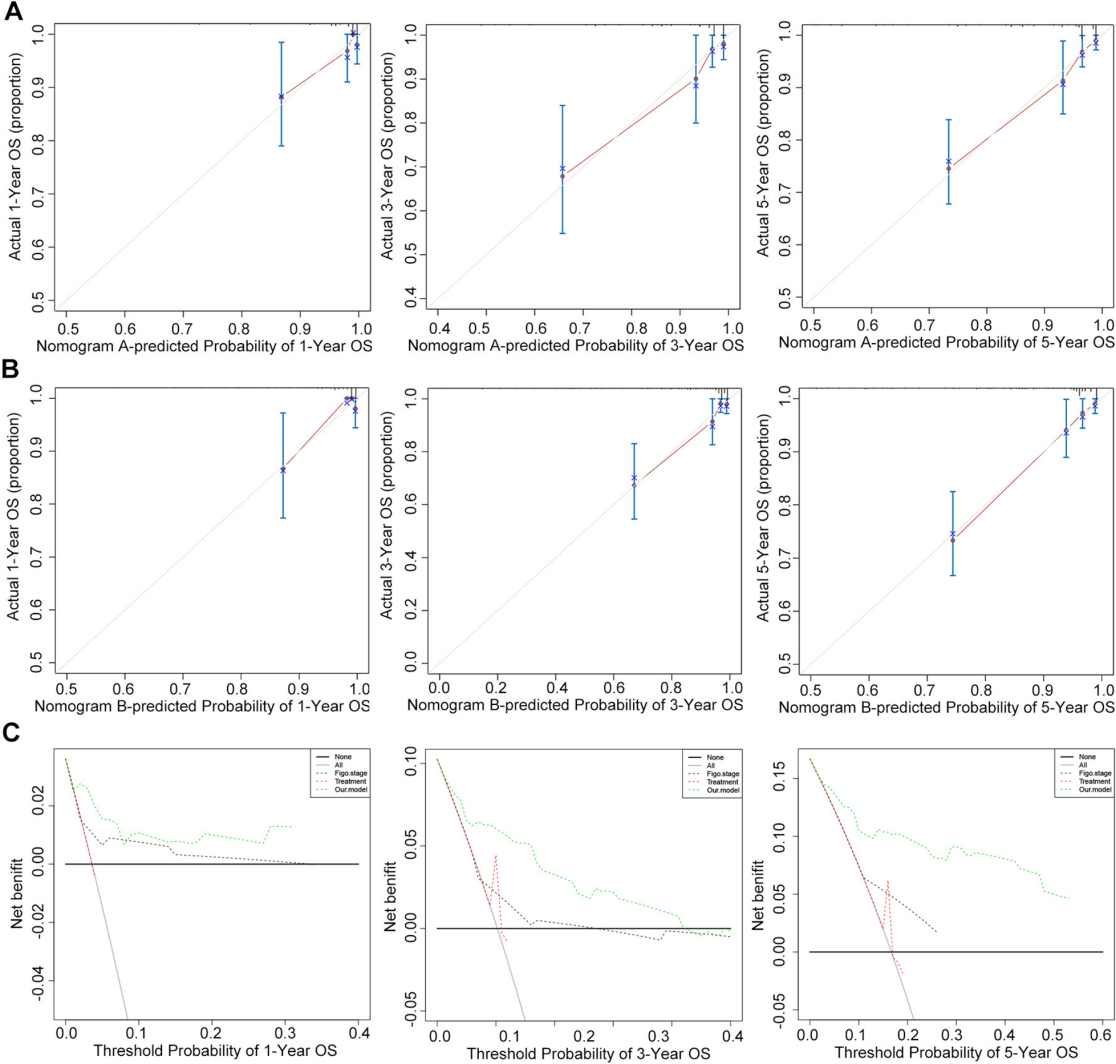
The calibration curves and decision curve analysis for predicting patients’ overall survival (OS). The calibration curves for predicting OS at 1, 3, and 5 years in all cases. **A**, **B** Nomograms A and B, with the model-predicted OS plotted on the x-axis and actual OS plotted on the y-axis. Closer alignment with the diagonal line represents better estimation. **C** Decision curve analysis for 1-, 3-, and 5-year survival predictions

Two different nomograms that predict the OS of ECA patients were built and compared for efficiency. Nomogram A included age, HPV E6/E7 RNAscope, LVI, and LNI, while nomogram B included age, HPV E6/E7 RNAscope, LVI, LNI, FIGO stage, and treatment. The results of time-dependent ROC curve for OS showed that AUCs of nomogram A and FIGO stage were higher than treatment (Fig. 3A). The resulting variables from the Cox proportional analysis were used to build the prognostic nomograms for OS (Fig. 3B and C). Each prognostic factor within the nomogram was assigned a point. By sum of the total points from all variables combined with the location the total point scale allowed us to obtain the probabilities of the outcomes by drawing a vertical line towards the axis labeled “1-, 3-, 5-Year survival probability” (Fig. 3B and C). The results of the comparison of efficiency between our nomogram and the conventional systems are shown in Table 4. In the two nomograms, no significant difference was observed (C-index: 0.825, 95% CI = 0.754–0.896 vs. 0.836, 95% CI = 0.771–0.902). The C-index of nomogram A (0.825, 95% CI = 0.754–0.896) was better than those of the FIGO system (0.653, 95% CI = 0.567–0.740) and treatment (0.578, 95% CI = 0.506–0.651). In Fig. 4A and B, the calibration plot for the OS rates for 1, 3, and 5-years was in line with both the nomogram and the actual observation. Besides, our model seemed to have a higher prediction accuracy (Fig. 4C).

**Table 4:** The C-index of our model, FIGO stage, Treatment for prediction of OS in the ECA

Factors	C-index	95 CI%	*P*
Nomogram A	0.825	0.754–0.896	
FIGO stage	0.653	0.567–0.740	
Treatment	0.578	0.506–0.651	
Nomogram B	0.836	0.771–0.902	
Nomogram A vs FIGO stage			**0.002**
Nomogram A vs Treatment			** < 0.001**
Nomogram A vs Nomogram B			0.139

Nomogram A: age + HPV RNA scope + LVI + LNI

Nomogram B: age + HPV RNA scope + LVI + LNI + FIGO stage + Treatment

C-index = concordance index; LVI, lymph vascular invasion; LNI, lymph node involvement; P values are calculated based on normal approximation using function rcorrp.cens in Hmisc package

### Risk stratification of prognosis

**Fig. 5:**
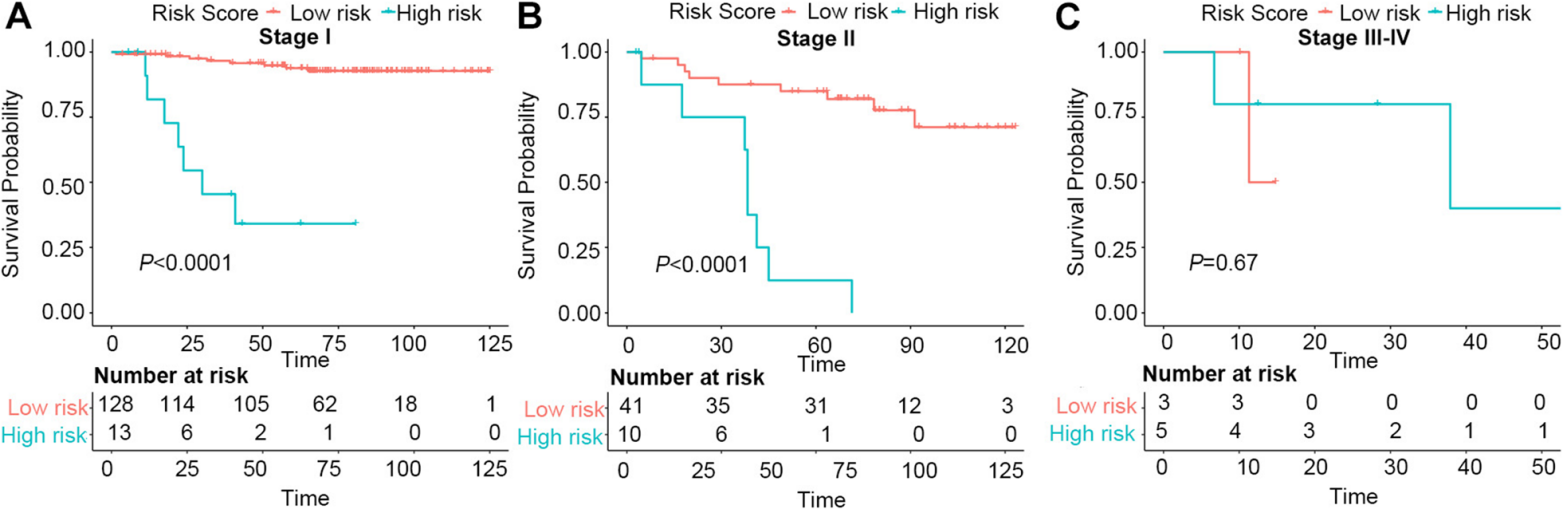
Graphs showing the Kaplan–Meier curves of both groups based on the predictors from the nomogram model. **A** stage I. **B** stage II. **C** stages III and IV

All patients were divided into a low (≤ 15.75) and a high-risk group (> 15.75) for OS (Additional file 1: Fig. S2B). The 1,3, and 5-year OS rates were 94.2%, 84.3%, 70.3%, respectively, in the low-risk group and 71.4%, 46.4%, 10.7%, respectively, in the high-risk group (Table 5). Moreover, significant differences in OS were noted between patients with stage I/II ECA and those with III/IV ECA (Fig. 5). Each risk subgroup represented a distinct prognosis, and our system accurately separated the OS rates of the two subgroups.

**Table 5:** Point assignment and prognostic score of the nomogram model

Variables	Prognostic Score	1-Year OS (%)	3-Year OS (%)	5-Year OS (%)
Age (years)				
≤ 37	7.5	11.8	26.5	35.3
> 37	0	1.8	6.0	10.8
HPV RNA scope				
≤ 3.3	7	5.1	13.1	21.2
> 3.3	0	2.0	5.9	8.9
Lymph nodes invasion				
No	0	1.9	4.5	7.1
Yes	8.25	8.7	26.1	41.3
Lymph vascular invasion				
None	0	1.4	6.5	10.1
Focal	3.25	2.7	10.8	13.5
Moderate	6.5	13.3	20.0	26.7
Extensive	9.75	20.0	30.0	70.0
Total prognostic score				
≤ 15.75		94.2	84.3	70.3
> 15.75		71.4	46.4	10.7

## Discussion

Our study is the first of its kind to describe a prognostic nomogram model for invasive ECA in a large Chinese series by reporting HPV E6/E7 RNAscope using a new RNA ISH assay that recognizes 18 high-risk HPV types. The most important finding of our research are as follows: (1) the validity of the HPVA and NHPVA categories were supported by p16 immunophenotyping and HPV status; (2) HPV E6/E7 RNAscope is more sensitive and specific than p16 and HPV genotype in identifying HPVA; (3) we established a prognostic nomogram model based on ECA pathogenesis that adjusted for age, LVI, HPV E6/E7 RNAscope, and LNI, which had a greater predictive efficiency than the current conventional staging system; (4) in the future, we believe, based on our model, ECA patients could be divided into low and high-risk groups.

HPV E6/E7 RNAscope measurement is not routinely available in most hospitals; hence the methodology cannot be extrapolated to standard practice. About 94% of adenocarcinomas are associated with HPV, particularly high risk strains like 16 and 18 [5], which is consistent with our results. In the present study, 87% (160/184) of HPVA samples overexpressed p16 or were HPV positive by RNAscope, validating the IECC criteria and previous studies [9, 20]. In terms of the IECC criteria, the HPV RNAscope is more specific for clinical practice, and it is more robust than p16 IHC, HPV DNA, and HPV genotype in identifying HPVA. Despite this, occasional p16 and/or HPV negative HPVAs were also identified. When outlying cases were excluded from the statistical analysis, 25 of the 184 HPVA cases were p16- and HPV-positive. All HPVA cases in the present study were systematically reviewed. The specimens were more than 5 years old and may not have been optimal tissues for the performance of HPV RNAscope and p16. A recent study reported that the HPV associated ECA might represent unusual morphological variants of gastric-type carcinoma [21]. Furthermore, rare HPV genotypes not included in the RNA ISH probe set may be responsible for the negative HPV results. There are precedents in the literature for p16- and HPV-associated neoplasia. Notably, p16-positive invasive squamous carcinomas of the cervix have been reported [22] to be closely associated with methylation-induced inactivation of the p16 gene and allelic loss of p16 [23, 24]. Other studies have reported that the HPV genome can be differentially expressed with varying heterogeneity between the primary and the metastatic sites suggesting that some HPV-related usual-type adenocarcinomas could have detectable HPV in multiple sections of primary carcinoma; hence all metastatic sites should be tested [20, 25, 26].

Clinicopathological variables carry a prognostic significance and usually affect clinical management [27, 28]. Depending on variables such as LVI and desire for fertility, histological-based risk evaluation should be considered during tissue sampling at initial diagnosis [15, 29]. The National Comprehensive Cancer Network guidelines recommend chemotherapy or radiotherapy as the standard treatment for patients with advanced ECA, hence, the performance of nomograms must be examined separately in patients treated using these therapies. The C-indices of nomograms A and B for predicting OS in treated patients were significantly higher than those of conventional classification, indicating that nomograms still have significant clinical value in patients with ECA. With the addition of the FIGO stage and treatment into nomogram B, the added values of these parameters over nomogram A were 0.836 and 0.825, respectively, across the entire population. There were no significant differences between nomograms A and B, probably due to the small sample size of patients with advanced-stage ECA.

The present study had several limitations. Firstly, there may have been selection bias in our patient cohort. Secondly, we lacked data on the impact of our nomogram on other prognostic endpoints, such as disease-free survival (DFS) prediction. Thirdly, in situ measurement of HPV RNA using RNAscope is not easily available and not globally standardized. Fourthly, our sample size was relatively small and from only one center. In order to validate our results, a study with a larger sample with multicenter data is required. Lastly, it remains unclear whether our nomogram can be applied to ECA patients with advanced-stage that is, stage III and IV.

## Conclusions

We generated new nomograms that incorporate HPV E6/E7 mRNA in situ hybridization and patients’ clinical characteristics in order to prognosticate the OS rate in ECA patients. Our simple and explicit nomograms have a good clinical application value, with a good diagnostic discernment and calibration ability. They may be a useful tool for assessing the prognosis and the management of ECA patients.

## Supplementary Information

**Additional file 1: Figure S1**. Representative images for clinicopathological features in paraffin-embedded endocervical adenocarcinoma (ECA) samples. **Figure S2.** The best cutoff values for all variables as determined by using X-tile in all cases of endocervical adenocarcinoma (ECA). **Figure S3.** Endocervical adenocarcinoma (ECA): proportions of combinations of positive human papillomavirus (HPV) tests and p16 immunohistochemistry (IHC). **Figure S4.** The positive rates of human papillomavirus (HPV) subtypes, mRNA, protein, and p16 protein in the subgroups of endocervical adenocarcinoma (ECA) cases.

**Additional file 2: Table S1.** The positive proportion of factors examined in ECA samples. **Table S2.** Correlation of P16 IHC, HPV DNA, HPV genotype and HPV RNAscope. **Table S3.** HPV genotypes in different ECA histological types.

## Data Availability

The authenticity of this article has been validated by uploading the key raw data onto the Research Data Deposit public platform (www.researchdata.org.cn) with the approval RDD number RDDA2020001638.

## Abbreviations

HPV: Human papillomavirus
HPV E6/E7 RNAscope: Human papillomavirus E6/E7 mRNA in situ hybridization;
ECA: Endocervical adenocarcinoma
C-index: Concordance index
HPVA: HPV-associated adenocarcinoma
NHPVA: Non-HPV-associated adenocarcinoma
OS: Overall survival
LVI: Lymphovascular invasion
LNI: Lymph node involvement
