# Supplementary material for: HPV E6/E7 mRNA in situ hybridization in endocervical adenocarcinoma: implications for prognosis and diagnosis
Source: Cancer Cell Int. 2021 Dec 3;21:643. doi: 10.1186/s12935-021-02349-1 (PMC8641184; doi:10.1186/s12935-021-02349-1)
Supplement: Supplementary file 1 — Additional file 1: Figure S1. Representative images for clinicopathological features in paraffin-embedded endocervical adenocarcinoma (ECA) samples. Figure S2. The best cutoff values for all variables as determined by using X-tile in all cases of endocervical adenocarcinoma (ECA). Figure S3. Endocervical adenocarcinoma (ECA): proportions of combinations of positive human papillomavirus (HPV) tests and p16 immunohistochemistry (IHC). Figure S4. The positive rates of human papillomavirus (HPV) subtypes, mRNA, protein, and p16 protein in the subgroups of endocervical adenocarcinoma (ECA) cases. [file 12935_2021_2349_MOESM1_ESM.docx]

**
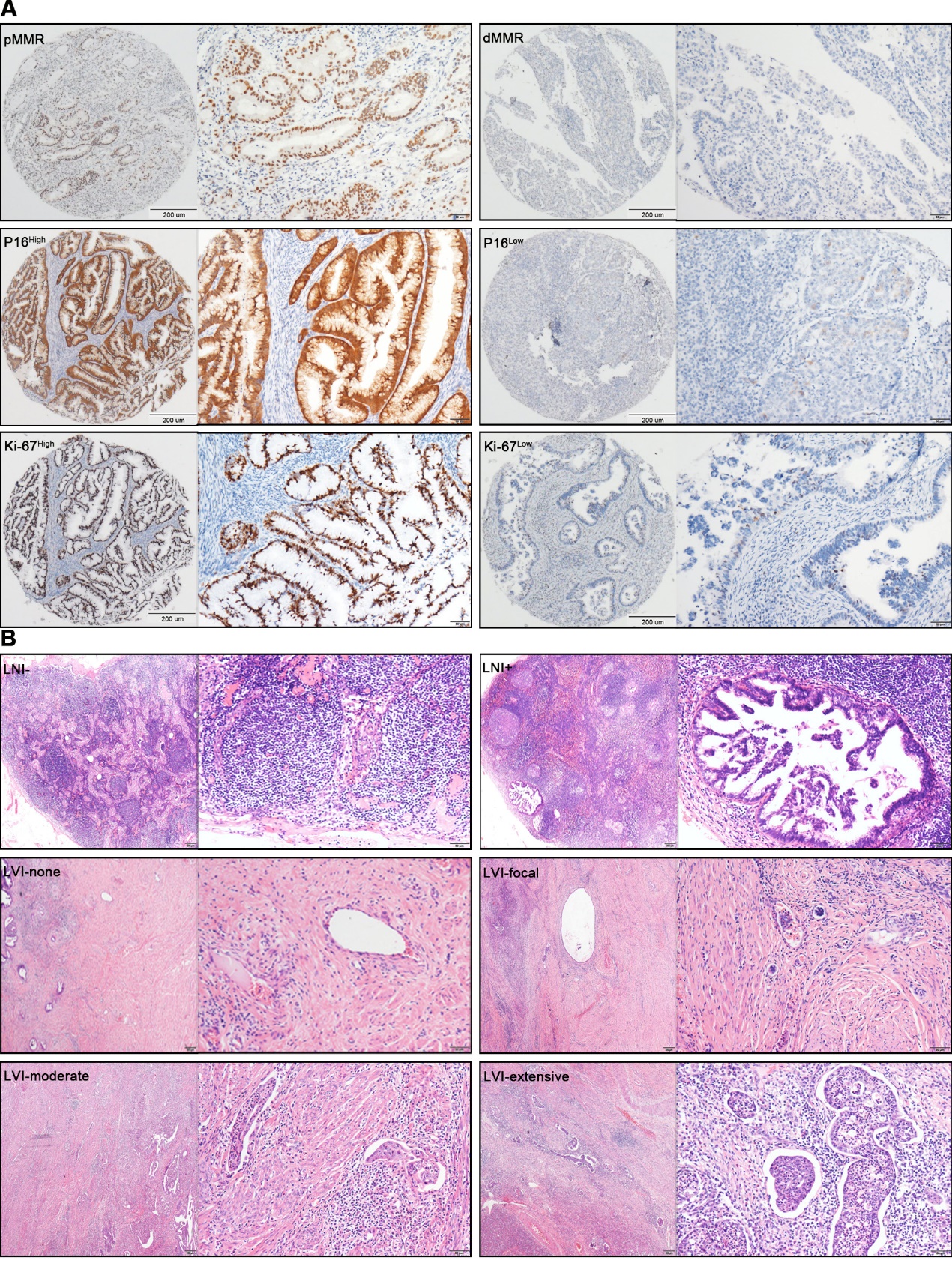
**

**Figure S1. Representative images for clinical pathologic features in paraffin-embedded endocervical adenocarcinoma (ECA) samples.** (A) Representative immunohistochemistry images for pMMR, dMMR, p16 high, p16 low, Ki-67 high, and Ki-67 low are shown. (B) Representative hematoxylin and eosin images for lymph node involvement (LNI)-negative, LNI-positive, lymphovascular invasion (LVI)-none, LVI-focal, LVI-moderate, and LVI-extensive are shown.

**
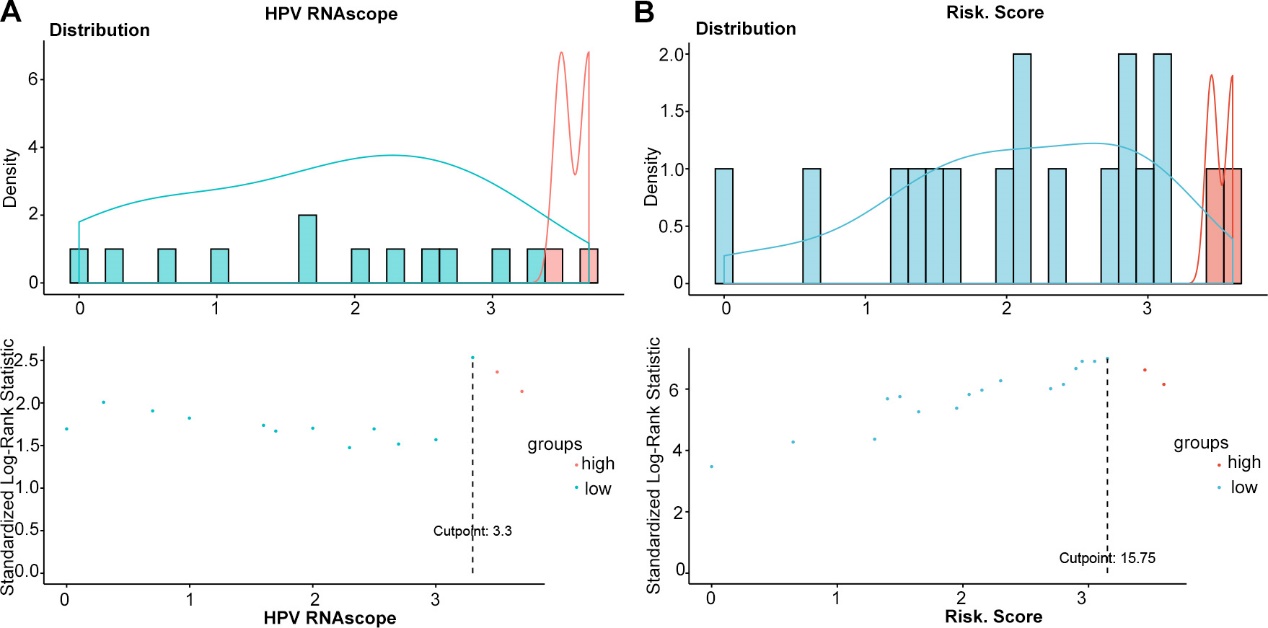
**

**Figure S2. The best cutoff values for all variables determined using X-tile in all cases of endocervical adenocarcinoma (ECA).** (A) The best cutoff values for human papillomavirus E6/E7 RNAscope were determined by X-tile. (B) The best cutoff values for the low-risk and high-risk groups were determined by X-tile.


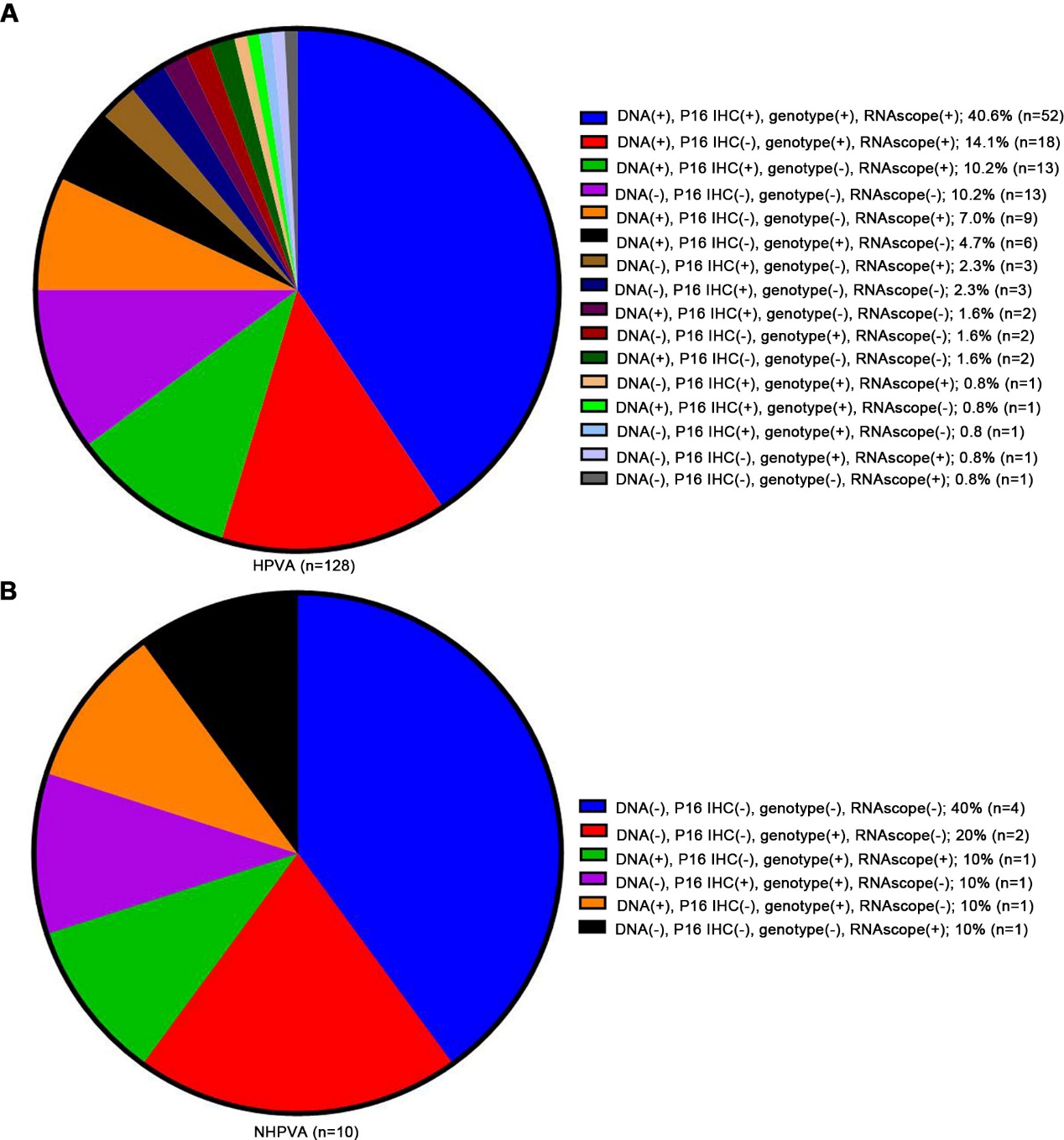


**Figure S3. Endocervical adenocarcinoma (ECA): proportions of combinations of positive human papillomavirus (HPV) tests and p16 immunohistochemistry (IHC).** The positive rates of HPV tests and p16 IHC in HPV-associated (A) and non-HPV-associated (B) cases.


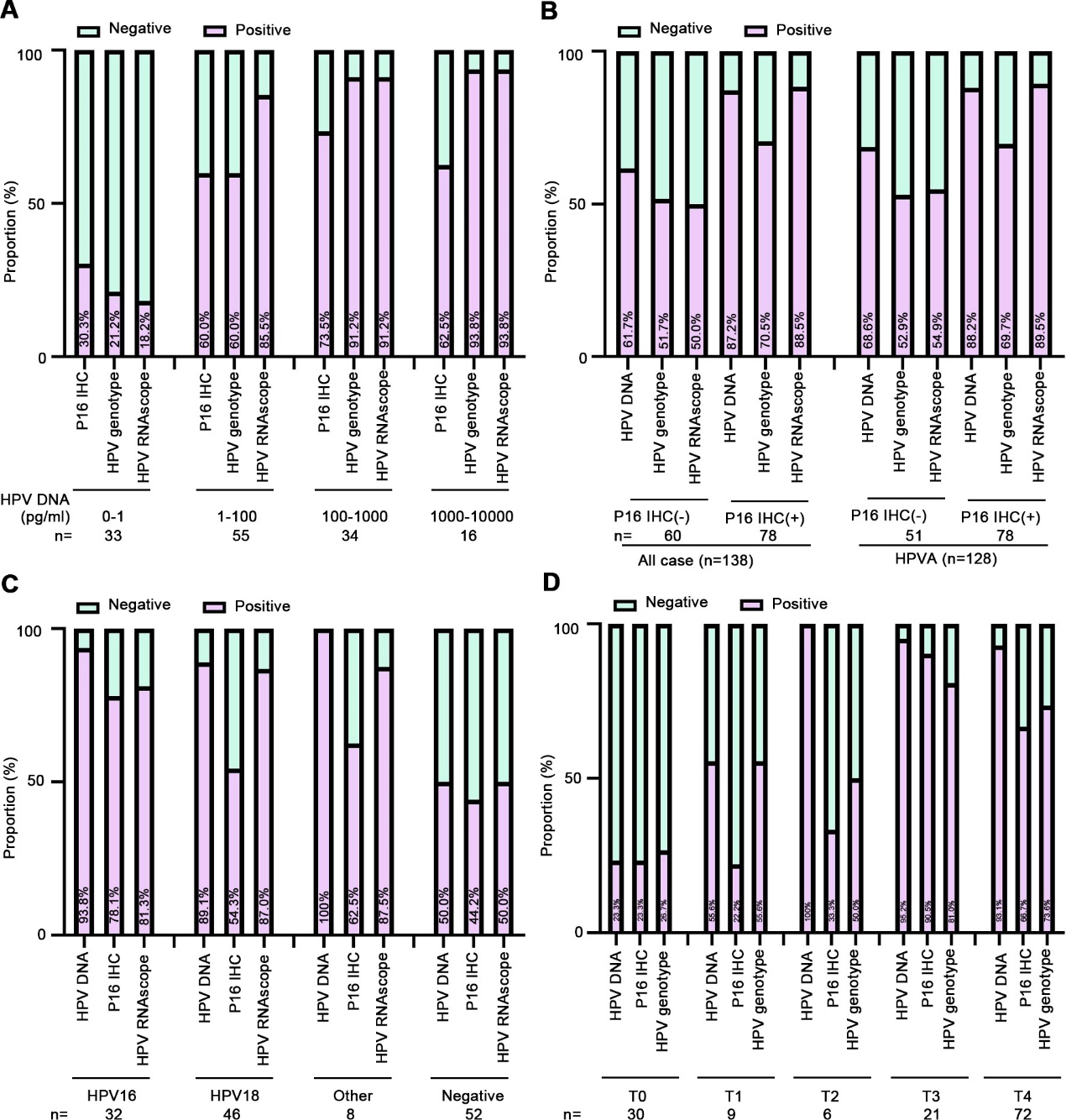


**Figure S4. The positive rates of human papillomavirus (HPV) subtypes, mRNA, protein, and p16 protein in the subgroups of endocervical adenocarcinoma (ECA) cases.** (A) Positive rates of HPV subtypes, mRNA, and p16 protein in ECA cases with different serum HPV levels. (B) Positive rates of HPV DNA, genotypes, and mRNA in all cases and in HPV-associated cases with different p16 expression. (C) Positive rates of HPV DNA, mRNA and p16 protein in ECA cases with different HPV subtypes. (D) The positive rates of HPV DNA, mRNA, and p16 protein in ECA cases with different HPV mRNA; p16 protein in tissues was detected using immunohistochemistry.
