# Supplementary material for: HPV E6/E7 mRNA in situ hybridization in endocervical adenocarcinoma: implications for prognosis and diagnosis
Source: Cancer Cell Int. 2021 Dec 3;21:643. doi: 10.1186/s12935-021-02349-1 (PMC8641184; doi:10.1186/s12935-021-02349-1)
Supplement: Supplementary file 2 — Additional file 2: Table S1. The positive proportion of factors examined in ECA samples. Table S2. Correlation of P16 IHC, HPV DNA, HPV genotype and HPV RNAscope. Table S3. HPV genotypes in different ECA histological types. [file 12935_2021_2349_MOESM2_ESM.docx]

| **Table S1. The positive proportion of factors examined in ECA samples.** | | |
| --- | --- | --- |
|  | **HPVA** | **NPHVA** |
| P16 IHC | 62.2% (115/185) | 20% (3/15) |
| HPV DNA | 80.6% (108/134) | 20% (2/10) |
| HPV genotype | 64.6% (115/178) | 35.7% (5/14) |
| HPV RNAscope | 76.2% (141/185) | 20% (3/15) |

HPVA, HPV-associated adenocarcinoma; NHPVA, nonHPV-associated adenocarcinoma; IHC, immunohistochemistry.

| **Table S2. Correlation of P16 IHC, HPV DNA, HPV genotype and HPV RNAscope.** | | | | |
| --- | --- | --- | --- | --- |
|  |  | **HPV RNAscope** | |  |
| **Variable** | **Cases** | **Negative** | **Positive** | ***P* value** |
| **P16 IHC** |  |  |  | **<0.001** |
| Negative | 82 | 41(50%) | 41(50%) |  |
| Positive | 118 | 15(12.7%) | 103(87.3%) |  |
| **HPV DNA** |  |  |  | **<0.001** |
| Negative | 34 | 27(79.4%) | 7(20.6%) |  |
| Positive | 110 | 12(10.9%) | 98(89.1%) |  |
| Not available | 56 | 17(30.4%) | 39(69.6%) |  |
| **HPV genotype** |  |  |  | **<0.001** |
| Negative | 72 | 38(52.8%) | 34(47.2%) |  |
| Positive | 120 | 17(14.2%) | 103(85.8%) |  |
| Not available | 8 | 1(1.8%) | 7(4.9%) |  |

IHC, immunohistochemistry.

| **Table S3. HPV genotypes in different ECA histological types.** | |
| --- | --- |
| **Histological types (positive cases/total)** | **HPV genotypes detected by PCR** |
| HPVA (114/184) | 47ˣHPV16, 56ˣHPV18, 2ˣHPV45, 3ˣHPV(16+18), 2ˣHPV(18+45), 2ˣHPV(53/56/66), 1ˣHPV(18+39/59/68), 1ˣHPV(26/51/82) |
|  | 63 negative |
|  | 7 not available |
| NHPVA (6/16) | 2ˣHPV16, 4ˣHPV18 |
|  | 9 negative |
|  | 1 not available |

HPVA, HPV-associated adenocarcinoma; NHPVA, non HPV-associated adenocarcinoma.
